# Supplementary material for: PMI-controlled mannose metabolism and glycosylation determines tissue tolerance and virus fitness
Source: Nat Commun. 2024 Mar 8;15:2144. doi: 10.1038/s41467-024-46415-4 (PMC10923791; doi:10.1038/s41467-024-46415-4)

Supplementary Information

For

PMI-controlled mannose metabolism and glycosylation determines tissue tolerance and virus fitness

Liang et al.,

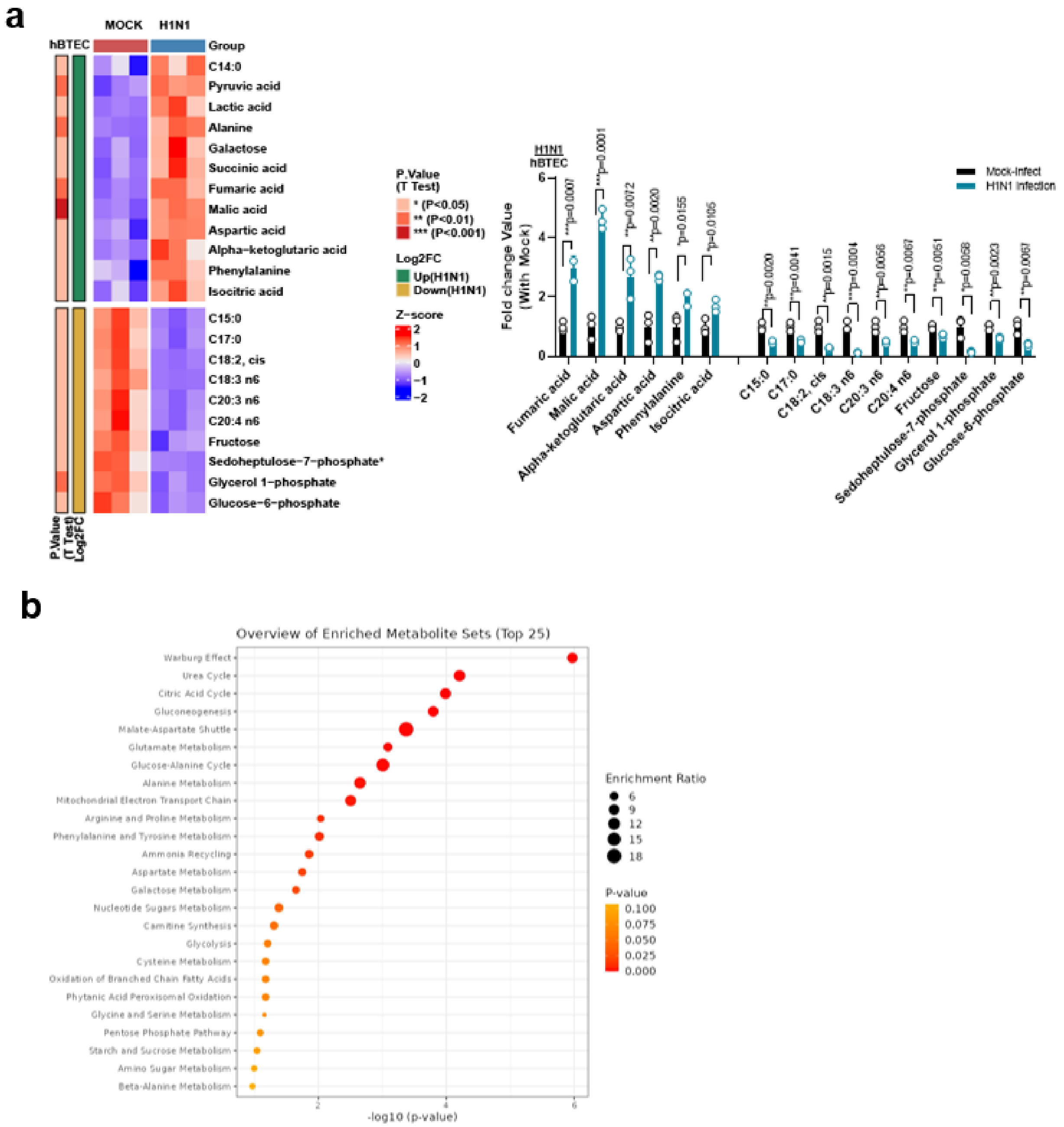

Supplementary Figure 1. Metabolomic profiling of human bronchial/tracheal epithelial cells (hBTECs) after H1N1 infection. hBTECs were infected with H1N1(5MOI). After 12h, cell lysates were harvested for targeted metabolomics by LC-MS/MS analysis. **(a)** Heatmap and fold change (FC) of metabolites comparing infection with non-infection groups (n=3 biological repeats). ****P*<0.001, ** *P* >0.01 and * *P* <0.05 by Student's *t* test. **(b)** Metabolite set enrichment analysis showing the top 25 enriched pathways upon H1N1 infection. The results were analyzed with MetaboAnalyst 5.0.

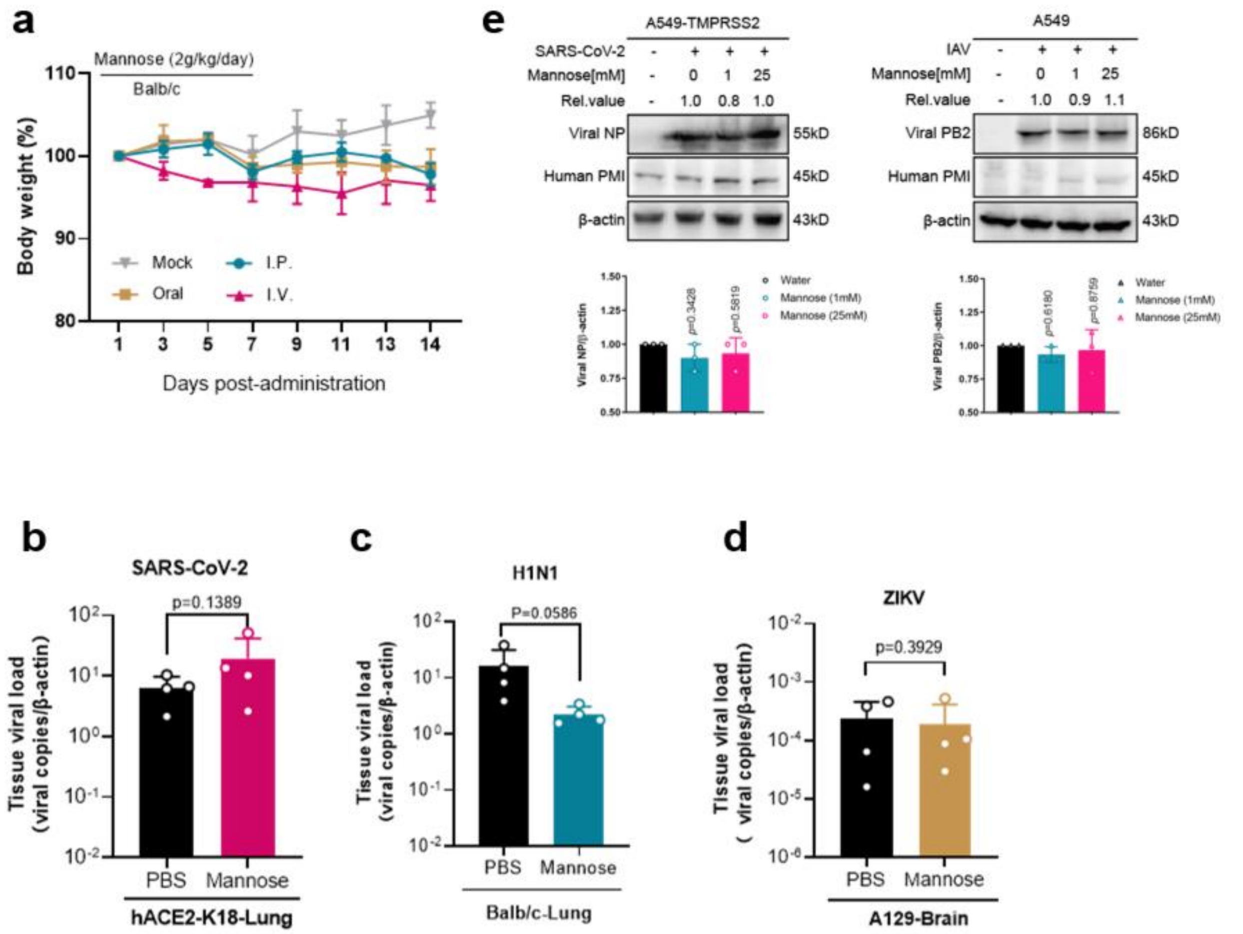

Supplementary Figure 2. In vivo toxicity and antiviral activity of mannose monotherapy. (a) Body weight change of BALB/c mice after mannose (2g/kg/day) administration for consecutive 14days. Mannose were delivered via intraperitoneal injection (I.P.), or oral gavage (P.O.), or intravenous injection (I.V.). Body weight of each group (n=5 mice/group) was recorded every other day until day 14. **(b-d)** Change of tissue viral load after mannose treatment (corresponding to main Figures 1b to 1d). Viral load was measured by RT-qPCR method in SARS-CoV-2 infected mouse lungs, H1N1-infected mouse lungs, and ZIKV-infected mouse brains in different animal models as indicated. Unpaired student *t* test was used (n=4 biological repeats). **(e)** SARS-CoV-2-infected A549-ACE2-TMPRSS2 cells (0.1MOI, 24hpi, left panel) and H1N1-infected A549 cells (0.1MOI, 24hpi, right panel) were treated with mannose at the indicated concentrations. The cell lysates were lysed by RIPA buffer and subject to viral antigen detection (SARS-CoV-2-NP and H1N1-PB2) by Western blotting. Relative value indicates the normalized signal intensity between viral antigens and house-keeping β-actin expression. Three independent repeat experiments were performed and analyzed using One-way ANOVA with Dunnett's post-hoc test when compared with the water group. Shown are one representative result.

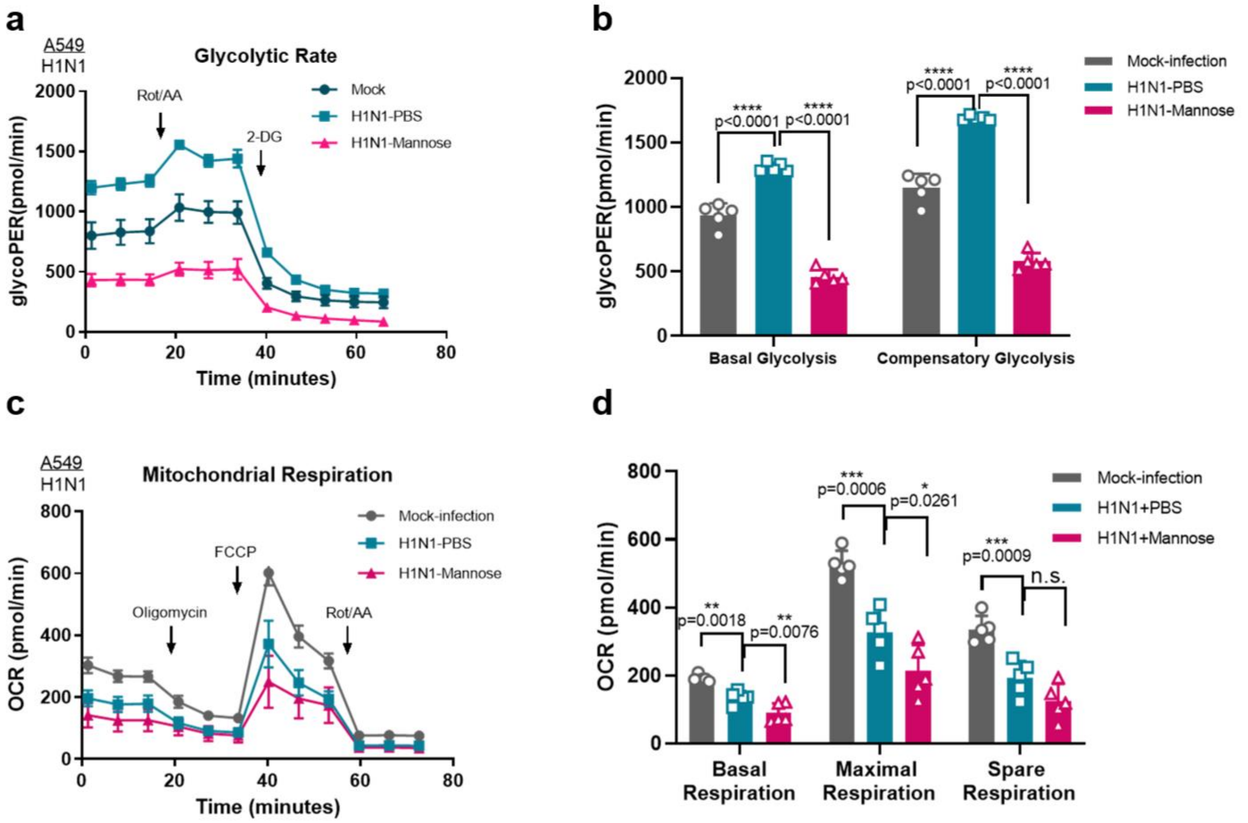

Supplementary Figure 3. Bioenergetic monitoring of H1N1-infected A549 cells after mannose treatment. (a) Bioenergetic monitoring of H1N1-infected A549 cells after mannose treatment. Cells were pre-treated with mannose (25mM) or PBS for overnight before virus infection (MOI=2) for another 12h. Cells were analyzed using a Seahorse XF analyzer to determine the protein efflux rate derived from glycolysis (glycoPER). The changes in glycoPER for the time indicated were triggered by the addition of Rot/AA (0.5 μM and 50 mM 2-DG, respectively. The results are shown as mean ± SD. **(b)** Basal and compensatory glycolysis ratios after mannose treatment. The results are normalized with 10^4 cells via Hoechst 33342 staining. One-way ANOVA with Dunnett's post-hoc test when compared with the H1N1-PBS control group. **(c)** Cells were analyzed using a Seahorse XF analyzer to determine the oxygen consumption rates (OCR) as reflection of mitochondrial respiration. Oligomycin (1μM), FCCP (1μM), and Rot/AA (0.5μM) were used, respectively. **(d)** Basal, maximal, and spare mitochondrial respiration ratios after mannose treatment. The results are normalized with 1E4 cells via Hoechst 33342 staining. One-way ANOVA with Dunnett's post-hoc test when compared with the H1N1+PBS control group (n=5 biological repeats). ****P<0.0001, *** P<0.001, ** P<0.01, * P<0.05, and n.s. indicates non-significant.

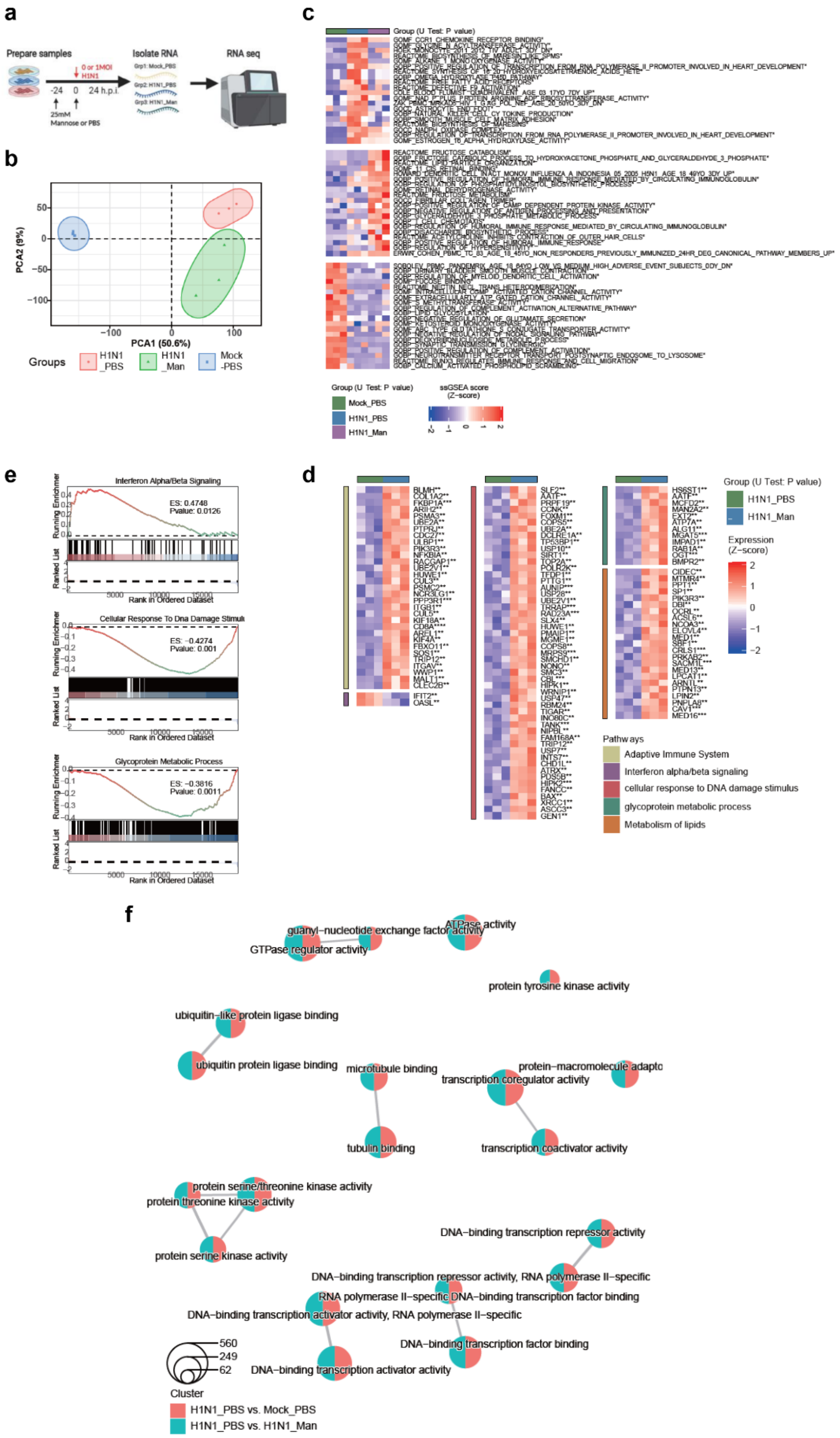

Supplementary Figure 4. Transcriptomic landscape of human lung epithelial A549 cells after H1N1 infection and mannose treatment. (a) Scheme of the transcriptomic study on H1N1-

infected A549 cells treated with or without mannose. MOI, the multiplicity of infection; hpi, hours post infection. **(b)** Principal components analysis of RNA-seq dataset after ‘reads per kilobase of transcript per million reads mapped’ normalization on each gene expression level. Each dot represents one sample. The percentage labelled on the x or y axis represents the proportion of variance explained with each principal component (PC). **(c)** Top-enriched pathways of significantly upregulated genes (FDR < 0.05, fold change > 2) compared between mock-infected cells versus H1N1 infection treated with PBS and versus H1N1 infection treated with mannose. Pathway analysis was performed by Metascape. **(d)** Heat map of the genes enriched in the adaptive immune system, interferon alpha/beta signaling, cellular response to DNA damage stimulus, glycoprotein metabolic process, and metabolism of lipids. These genes are changed significantly (fold change >2, FDR < 0.05) by mannose treatment, when compared to the H1N1-PBS group. **(e)** Gene set enrichment analysis (GSEA) enrichment plots of interferon alpha/beta signaling, cellular response to DNA damage stimulus, as well as glycoprotein metabolic process, are shown. **(f)** Network of enriched terms represented as pie charts between H1N1-infection versus Mock-infection, H1N1-infection versus H1N1-infection with mannose treatment. Pies are color-coded on the basis of the identities of the gene lists.

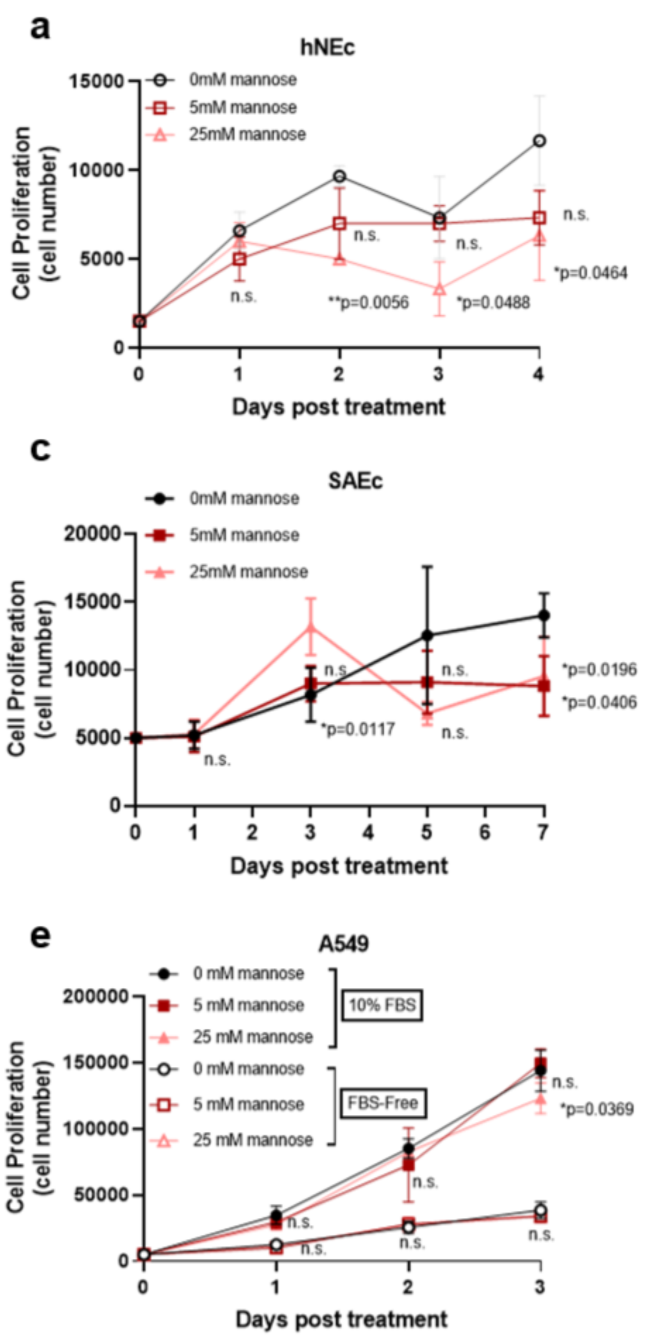

Supplementary Figure 5. Effects of mannose on cell proliferation and viability. (a) Growth curves of hNEs supplemented with 0, 5, or 25 mM of mannose. The results are shown as mean ± SD and analyzed by one-way ANOVA with Dunnett's post-hoc test when compared with the 0mM mannose control group. **(b)** Cell viability of hNEs supplemented with 0, 5, or 25 mM of mannose. Viability indicates the percentage of live cells out of the total number of cells and is normalized with the 0mM mannose control group. **(c)** Growth curves of SAEc supplemented with 0, 5, or 25 mM of mannose. The results are shown as mean ± SD and analyzed by one-way ANOVA with Dunnett's post-hoc test when compared with the 0mM mannose control group. **(d)** Cell viability of SAEc supplemented with 0, 5, or 25 mM of mannose. Viability indicates the percentage of live

cells out of the total number of cells and is normalized with the 0mM mannose control. **(e)** Growth curves of A549 supplemented with 0, 5, or 25 mM of mannose in the normal culture condition and Mock (FBS-free) condition. All results are shown as mean ± SD and analyzed by one-way ANOVA with Dunnett's post-hoc test when compared with the 0mM mannose control group (n=4 biological repeats). ** *P*<0.01, * *P* <0.05, and n.s. indicates non-significant.

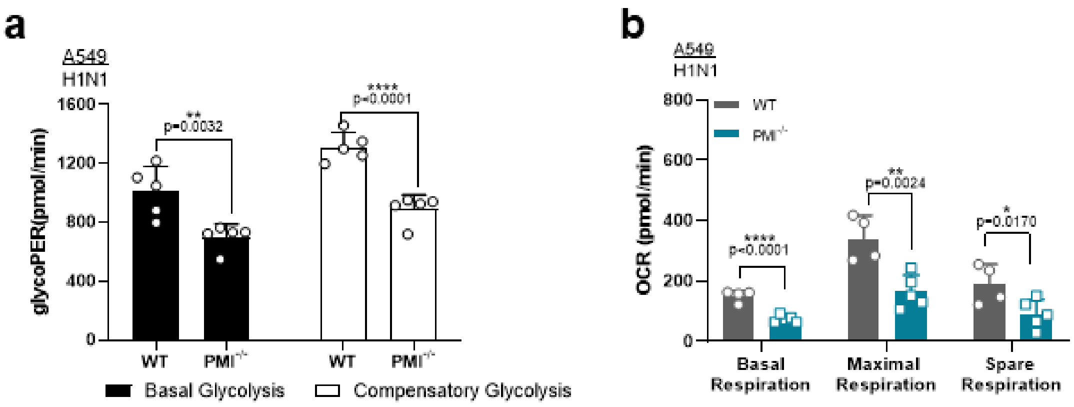

Supplementary Figure 6. Both mannose treatment and PMI depletion suppress glycolysis and downstream mitochondrial respiration. A549-WT cells and A549-PMI$^{-/-}$ cells were pre-treated with PBS before H1N1 infection (2MOI). Twelve hours after H1N1 infection, cells were subject to analysis by a Seahorse XF analyzer. **(a)** Basal and compensatory glycolysis ratios as determined by the glycoPER assay. **(b)** Basal, maximal, and spare mitochondrial respiration ratios as determined by the OCR assay. The results are normalized with those of mock-infected cells (n=5 biological repeats). Unpaired student *t* test among the indicated groups. ****P<0.0001, ** P<0.01, * P<0.05.

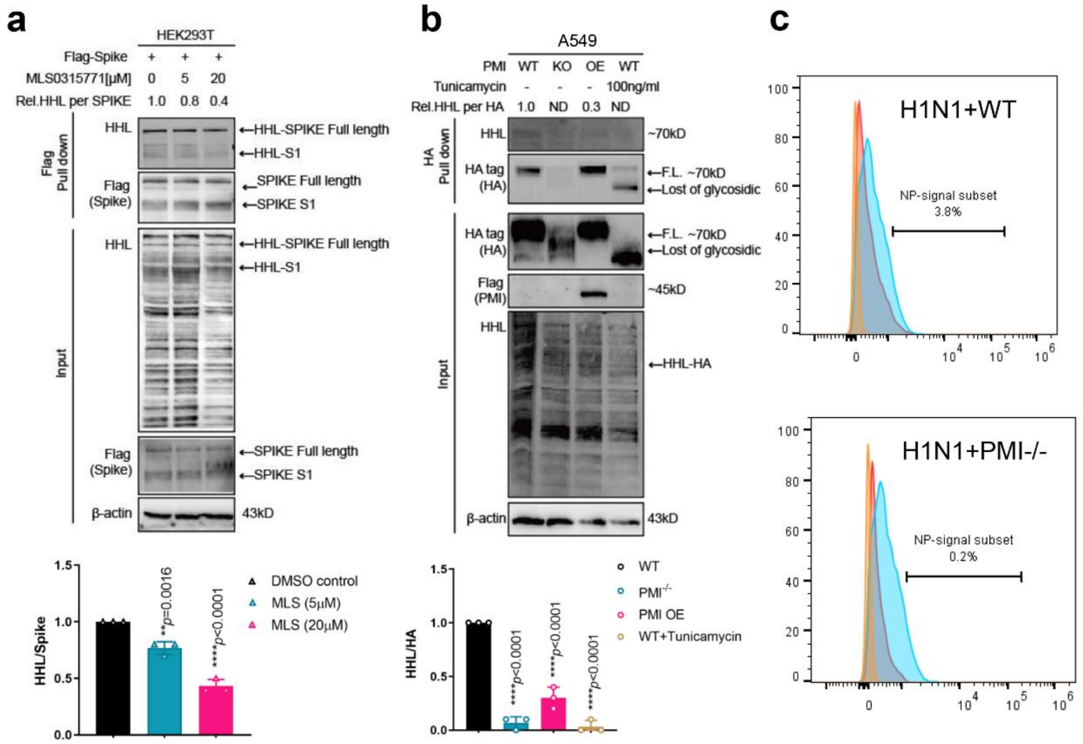

Supplementary Figure 7. PMI affect both SARS-CoV-2 spike and H1N1 HA protein glycosylation. (a) The PMI inhibitor MLS0315771 decreased the SARS-CoV-2 spike protein glycosylation. HEK293T cells were transfected with flag-spike constructs and treated with MLS0315771 for 24 hours before analysis of protein N-glycosylation. After pull-down experiment, full spectrum of host HHL glycosylation process particularly viral spike protein was analyzed by Western blot and lectin blot analyses. **(b)** HA tagged-viral hemagglutinin (HA) was overexpressed in WT, PMI$^{-/-}$, or PMI$^{-/-}$ rescued with exogenous PMI cells. Treatment with 100ng/ml tunicamycin was included as a positive control inhibitor of protein glycosylation. After 24 hours, pull-down experiments and HHL glycosylation analysis were performed similar as described in panel A. Relative value indicates the normalized signal intensity between HHL glycosylation level and IAV-HA expression, when compared with WT cells. The abbreviation 'ND' stands for 'not detected' and was assigned a value of 0 to enable statistical analysis. Three independent repeat experiments were performed for (**a)** and (**b)**, and analyzed using One-way ANOVA with Dunnett's post-hoc test when compared with DMSO or WT control group as indicated (n=3 biological repeats). ****P<0.0001, ** P<0.01. Shown are one representative results. **(c)** WT and PMI$^{-/-}$ cells were infected with H1N1 (0. 1MOI) for 2h before subject to anti-influenza-NP staining and flow cytometry. Shown are rates of NP-positive cells. The experiments are repeated twice for confirmation.

Supplementary Table

REAGENT or RESOURCE	SOURCE	IDENTIFIER
**Antibodies**		
Galanthus Nivalis Lectin (GNL), Fluorescein	Vector Labs	FL-1241-2
Goat anti-rabbit IgG (H+L) antibody conjugated to fluorescein isothiocyanate	Sigma–Aldrich	AP307F
Rabbit anti-β-actin	Invitrogen	PA5-78715
Rabbit anti-Influenza A H1N1 NA	Invitrogen	PA5-23363
Rabbit anti-DYKDDDDK Tag	Invitrogen	PA1-984B
Mouse anti-Phosphomannose Isomerase	Invitrogen	MA5-25979
Mouse anti-HIF1alpha	Invitrogen	MA1-516
Mouse anti-GAPDH	Invitrogen	MA1-16757
Goat anti-Rabbit IgG (H+L)	Invitrogen	31460
Goat anti-Rabbit IgG (H+L), Alexa Fluor® 488 conjugate	Invitrogen	A11008
Goat anti-Mouse IgG (H+L)	Invitrogen	31430
Goat anti-Mouse IgG (H+L), Alexa Fluor® 594 conjugate	Invitrogen	A11005
Rabbit anti-Hexokinase II	Abcam	ab209847
Rabbit anti-H1N1 Influenza A virus Nucleocapsid protein	Abcam	ab104870
Mouse anti-Influenza A Virus Hemagglutinin	Abcam	ab8262
**Plasmids and Virus Strains**		
MPI/Mannose Phosphate Isomerase cDNA ORF Clone	Sinobiological	HG17227-NF
human ACE2	Sinobiological	HG10108-CF

A/Hong Kong/415742/2009(H1N1) pdm09	In house	PMID: 20660098
SARS-CoV-2 strain B.1.1.7/Alpha	In house	GenBank accession number OM212469
Enterovirus A-71 (SZ/HK08-5)	In house	GenBank accession number GQ279369 .1
Zika virus (Puerto Rico strain PRVABC59)	Dr. Brandy Russell and Dr. Barbara Johnson, CDC, USA	
MERS-CoV (EMC/2012)	Dr Ron Fouchier (Erasmus Medical Center, the Netherlands)	
**Chemicals and drugs**		
Acrylamide/Bis-acrylamide, 30% solution	MERCK	A3574-5X
RNAiMAX Transfection Reagent	INVITROGEN	13778150
D-Fructose-6-phosphate disodium salt	MedChemExpress (New Jersey, USA)	HY-113407A
G418	MedChemExpress (New Jersey, USA)	HY-17561
Nirmatrelvir	MedChemExpress (New Jersey, USA)	HY-138687
Zanamivir	MedChemExpress (New Jersey, USA)	HY-13210
CCCP1	Sigma-Aldrich (Missouri, USA)	MAK148
Poly I:C	Sigma-Aldrich (Missouri, USA)	P9582
4′,6-diamidino-2-phenylindole	Sigma-Aldrich (Missouri, USA)	28718-90-3
PageRuler™ Prestained Protein Ladder, 10 to 180 kDa	Thermo	26616

PageRuler™ Plus Prestained Protein Ladder, 10 to 250 kDa	Thermo	26620
Pierce Phosphatase Inhibitor Mini Tablets, 20 tablets	Thermo	A32957
Pierce Protease and Phosphatase Inhibitor Mini Tablets, 20 tablets	Thermo	A32959
Pierce Protease and Phosphatase Inhibitor Mini Tablets,EDTA-free, 20 tablets	Thermo	A32961
Insulin	Sigma-Aldrich (Missouri, USA)	I-034
**Critical Commercial Assays**		
Succinate Assay Kit	Abcam	ab204718
IL-1β ELISA kit	Abclonal (Massachusetts, USA)	A16288
TNF-α ELISA kit	Abclonal (Massachusetts, USA)	A11534
Seahorse Cell Mito Stress Test Kit	Agilent	103015-100
Seahorse XF Glycolysis Stress Test Kit	Agilent	103015-100
CellTiter-Glo	Promega	G7571
Luciferase Reporter Assay System	Promega	E1501
RNeasy Mini Kit	Qiagen, Germantown, MD, USA	74004
MitoProbe™ JC-1 assay kit	Thermo	M34152
**Experimental Models: cell lines/animals**		
A549	ATCC®	CCL-185
Calu3	ATCC®	HTB-55™
Huh7	JCRB®	0403™
RD	ATCC®	CCL-136
U251	Sigma-Aldrich	09063001

MDCK	ATCC®	PTA-6503
VeroE6	ATCC®	CRL-1586™
HAP1-WT	Horizon Discoveries	
HAP1-PMI$^{-/-}$	Horizon Discoveries	
K18-hACE2 transgenic mice	CCMR, HKU	
BALB/c mice	CCMR, HKU	
IFNα/βR$^{-/-}$ (A129) mice	CCMR, HKU	
**Oligonucleotides**		
siPMI	Thermo Scientific	4427038
siScramble	Thermo Scientific	
Influenza A_M_F	IDT	CTTCTAACCGA GGTCGAAACG
Influenza A_M_R	IDT	GGCATTTTGGA CAAAKCGTCTA
human GAPDH_F	IDT	ATTCCACCCAT GGCAAATTC
human GAPDH_R	IDT	CGCTCCTGGAA GATGGTGAT
mouse GAPDH_F	IDT	AAGGTCATCCC AGAGCTGAA
mouse GAPDH_R	IDT	CTGCTTCACCA CCTTCTTGA
SARS-CoV-2 RdRp_F	IDT	CGCATACAGTC TTRCAGGCT
SARS-CoV-2 RdRp_R	IDT	GTGTGATGTTG AWATGACATGG TC
β-actin_F	IDT	ACGGCCAGGTC ATCACTATTG

β-actin_R	IDT	CAAGAAGGAAG GCTGGAAAAG
**Software / Equipment and others**		
MouseOx Plus	Starr Life Sciences Corp, USA	# 72-8091
XF96 Seahorse Biosciences Extracellular Flux Analyzer	Seahorse Bioscience (Agilent technologies)	N/A
CFX96	Bio–Rad, Berkeley, California, USA	N/A
Carl Zeiss LSM880 system	Dublin, CA, USA	N/A
Novoexpress	Seahorse Bioscience (Agilent technologies)	https://www.agilent.com/en/product/research-flow-cytometry/flow-cytometry-software/novocyte-novoexpress-software-1320805
Flowjo	BD biosciences	https://www.flowjo.com/
cellSens Imaging Software	Olympus-lifescience	https://www.olympus-lifescience.com/en/software/cellsens/

Uncropped scans of blots and gels in Supplementary Figures

Supplementary Figure 2e

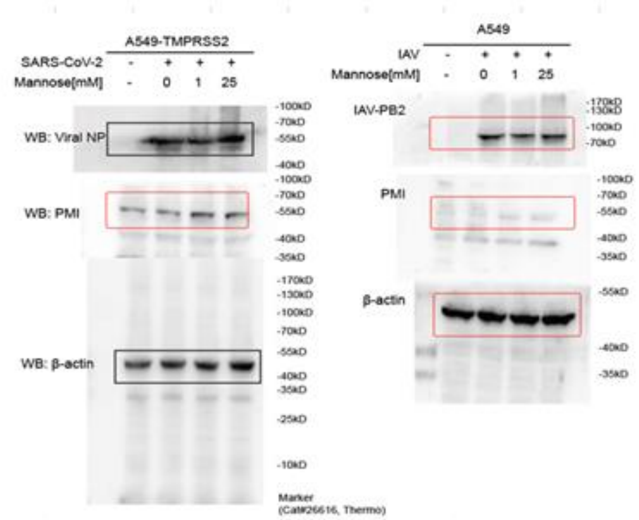

Supplementary Figure 7a

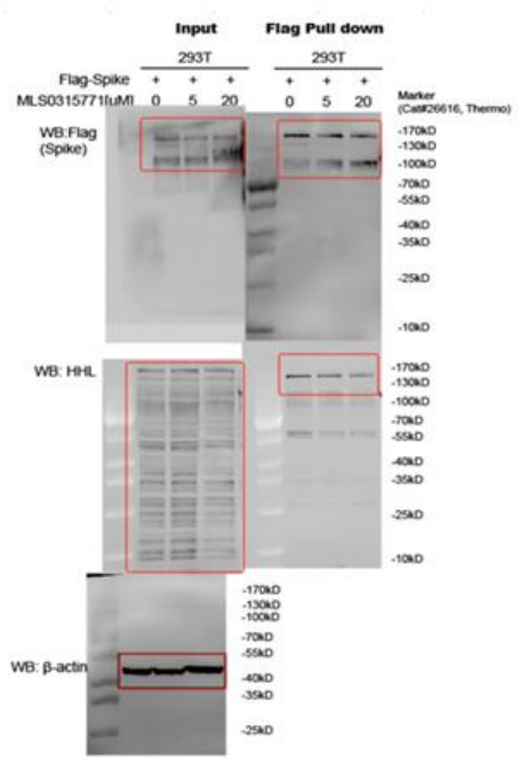

Supplementary Figure 7b

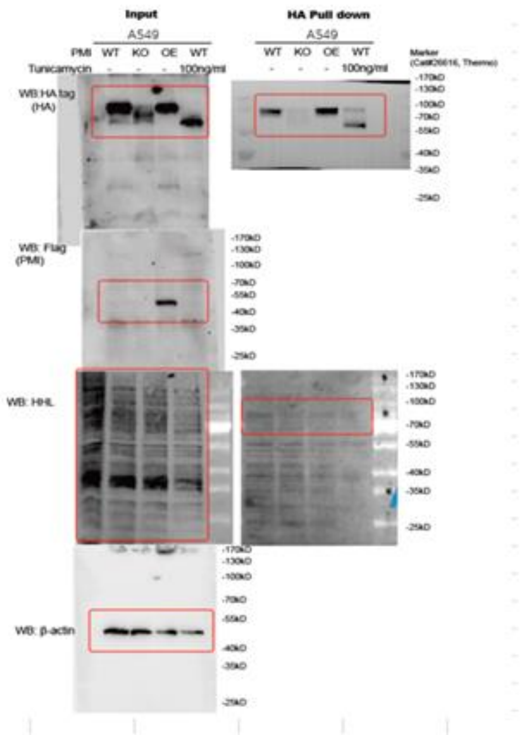

Supplement: Supplementary file 1 — Supplementary Information [file 41467_2024_46415_MOESM1_ESM.pdf]
