# Supplementary material for: PMI-controlled mannose metabolism and glycosylation determines tissue tolerance and virus fitness
Source: Nat Commun. 2024 Mar 8;15:2144. doi: 10.1038/s41467-024-46415-4 (PMC10923791; doi:10.1038/s41467-024-46415-4)
Supplement: Supplementary file 3 — Reporting Summary [file 41467_2024_46415_MOESM3_ESM.pdf]

nature portfolio

Corresponding author(s): Shuofeng Yuan

Last updated by author(s): Feb 7, 2024

Reporting Summary

Nature Portfolio wishes to improve the reproducibility of the work that we publish. This form provides structure for consistency and transparency in reporting. For further information on Nature Portfolio policies, see our Editorial Policies and the Editorial Policy Checklist.

Statistics

For all statistical analyses, confirm that the following items are present in the figure legend, table legend, main text, or Methods section.

n/a	Confirmed	
☐	☒	The exact sample size (*n*) for each experimental group/condition, given as a discrete number and unit of measurement
☐	☒	A statement on whether measurements were taken from distinct samples or whether the same sample was measured repeatedly
☐	☒	The statistical test(s) used AND whether they are one- or two-sided<br>*Only common tests should be described solely by name; describe more complex techniques in the Methods section.*
☒	☐	A description of all covariates tested
☒	☐	A description of any assumptions or corrections, such as tests of normality and adjustment for multiple comparisons
☐	☒	A full description of the statistical parameters including central tendency (e.g. means) or other basic estimates (e.g. regression coefficient) AND variation (e.g. standard deviation) or associated estimates of uncertainty (e.g. confidence intervals)
☐	☒	For null hypothesis testing, the test statistic (e.g. *F*, *t*, *r*) with confidence intervals, effect sizes, degrees of freedom and *P* value noted<br>*Give P values as exact values whenever suitable.*
☒	☐	For Bayesian analysis, information on the choice of priors and Markov chain Monte Carlo settings
☒	☐	For hierarchical and complex designs, identification of the appropriate level for tests and full reporting of outcomes
☒	☐	Estimates of effect sizes (e.g. Cohen's *d*, Pearson's *r*), indicating how they were calculated

Our web collection on statistics for biologists contains articles on many of the points above.

Software and code

Policy information about availability of computer code

Data collection	MouseOX Plus was used to collect vital signs data.<br>XF96 Seahorse Biosciences Extracellular Flux Analyzer was used to collect real-time metabolic data.<br>LightCycler96 (version SW11) was used to collect qPCR data.<br>AllianceQ9 software (v17-02) was used to collect Western blot data.<br>Carl Zeiss LSM880 was used to collect microscope data.<br>BD FACSAria SORP was used to collect flow cytometry data.<br>Agilent 7890B GC - Agilent 7010 Triple Quadrapole Mass Spectrometer system to collect metabolomics data.
Data analysis	The following software and websites were used for the analyses in this study: Agilent MassHunter Workstation. Quantitative Analysis Software (CPOS - PM Core, HKU), R package edgeR(v3.28.1), R package pheatmap (v1.0.12), R package ggplot2(v3.3.0), R package factoextra (1.0.7), DAVID server, GOseq R package,KOBAS software, Xcalibur(v4.0.27.19), MetaboAnalyst 5.0, Prism (v7.0), FlowJo (v10.0.7), BioRender.

For manuscripts utilizing custom algorithms or software that are central to the research but not yet described in published literature, software must be made available to editors and reviewers. We strongly encourage code deposition in a community repository (e.g. GitHub). See the Nature Portfolio guidelines for submitting code & software for further information.

Data

Policy information about availability of data

All manuscripts must include a data availability statement. This statement should provide the following information, where applicable:

- Accession codes, unique identifiers, or web links for publicly available datasets
- A description of any restrictions on data availability
- For clinical datasets or third party data, please ensure that the statement adheres to our policy

The raw RNA-seq data discussed in this manuscript have been deposited in Mendeley Data (doi:516 10.17632/gptx4bd7ss.1) and in GEO database with accession code GSE255604.
The raw metabolomics data has been deposited in MetaboLights under accession code MTBLS9368.
The raw metabolomics data performed in hBTEc model have been deposited in Mendeley Data (doi: 10.17632/z6fjtbrtn5.1).
The raw metabolomics data performed in human lung A549 model have been deposited in Mendeley Data (doi: 10.17632/7ky724g4jf.1).
The raw 13C metabolic flux analysis data performed in human lung A549 model have been deposited in Mendeley Data (doi: 10.17632/5z6cjhtfth.1).
Sequences of A/Hong Kong/415742/2009(H1N1)pdm09(HM100229-HM100236), SARS-CoV-2 HKU-001a(MT230904) , Enterovirus A-71 (GQ279369.1), and Zika virus (KU501215) are available through GenBank. Other supporting raw data are available from the corresponding author upon reasonable request. Source data are provided with this paper.

Research involving human participants, their data, or biological material

Policy information about studies with human participants or human data. See also policy information about sex, gender (identity/presentation), and sexual orientation and race, ethnicity and racism.

Reporting on sex and gender	Nil
Reporting on race, ethnicity, or other socially relevant groupings	Nil
Population characteristics	Nil
Recruitment	Nil
Ethics oversight	Nil

Note that full information on the approval of the study protocol must also be provided in the manuscript.

Field-specific reporting

Please select the one below that is the best fit for your research. If you are not sure, read the appropriate sections before making your selection.

☒ Life sciences ☐ Behavioural & social sciences ☐ Ecological, evolutionary & environmental sciences

For a reference copy of the document with all sections, see nature.com/documents/nr-reporting-summary-flat.pdf

Life sciences study design

All studies must disclose on these points even when the disclosure is negative.

Sample size	Sample size is chosen based on the standard of virus research as we previously published in Nature papers (doi: 10.1038/s41586-020-2577-1 and doi: 10.1038/s41586-021-03431-4). The sample size for each experiment is specified in each corresponding figure legend. A549, 293T-ACE2, Calu3, huh7, U251, RD, MDCK, Vero E6 cell lines were used in this study and n= at least 3 independent experiments were performed. For Human Small Airway Epithelial Cells (SAEc) ,Human Nasal Epithelial Cells (hNEc) and Human Bronchial/Tracheal Epithelial Cells experiments were performed with n= at least 3. For animal experiment, the principles of the 3Rs (Replacement, Reduction and Refinement) will be obeyed to balance the animal welfare and statistical power. For the animal study using golden Syrian hamsters for SARS-CoV-2 infection. Balb/c mice for IAV infection, A129 mice for ZIKV infection, a sample size of at least more than 5 was selected to evaluate the level of variation between individuals.
Data exclusions	No data has been excluded from the analyses presented in this manuscript.
Replication	As stated in the figure legends, the data are representative of two or three independent experiments with similar results.
Randomization	For all in vivo experiments, gender- and age- matched mice were randomized into different experimental groups. For the in vitro experiments, the same number of cells were seeded in each well of plates. The wells were randomly divided into treatment groups or control groups.
Blinding	The histopathologist was blinded for examining the lung histopathology of mice and hamster lungs. Blinding is not relevant to other experiments of the study. The experiments for different groups are carried out in parallel using the same set of protocols and the

experimental results are quantitative.

Reporting for specific materials, systems and methods

We require information from authors about some types of materials, experimental systems and methods used in many studies. Here, indicate whether each material, system or method listed is relevant to your study. If you are not sure if a list item applies to your research, read the appropriate section before selecting a response.

Materials & experimental systems

n/a	Involved in the study
☐	☒ Antibodies
☐	☒ Eukaryotic cell lines
☒	☐ Palaeontology and archaeology
☐	☒ Animals and other organisms
☒	☐ Clinical data
☒	☐ Dual use research of concern
☒	☐ Plants

Methods

n/a	Involved in the study
☒	☐ ChIP-seq
☐	☒ Flow cytometry
☒	☐ MRI-based neuroimaging

Antibodies

Antibodies used

Galanthus Nivalis Lectin (GNL), Fluorescein (Vector Labs ,FL-1241-2, 1:100 for Fluorescent-WB);
Goat anti-rabbit IgG (H+L) antibody conjugated to fluorescein isothiocyanate (Sigma–Aldrich ,AP307F, 1:1000 for WB);
Rabbit anti-β-actin (Invitrogen ,PA5-78715, 1:1000 for WB);
Rabbit anti-Influenza A H1N1 NA (Invitrogen ,PA5-23363, 1:1000 for WB);
Rabbit anti-DYKDDDDK Tag (Invitrogen ,PA1-984B, 1:1000 for WB);
Mouse anti-Phosphomannose Isomerase (Invitrogen ,MA5-25979, 1:1000 for WB);
Mouse anti-HIF1alpha (Invitrogen ,MA1-516, 1:1000 for WB);
Mouse anti-GAPDH (Invitrogen ,MA1-16757, 1:1000 for WB);
Goat anti-Rabbit IgG (H+L) (Invitrogen, 31460, 1:5000 for WB);
Goat anti-Rabbit IgG (H+L), Alexa Fluor®488 conjugate (Invitrogen ,A11008, 1:1000 for IF staining);
Goat anti-Mouse IgG (H+L) (Invitrogen ,31430, 1:1000 for WB);
Goat anti-Mouse IgG (H+L), Alexa Fluor®594 conjugate (Invitrogen ,A11005, 1:1000 for IF staining);
Rabbit anti-Hexokinase II (Abcam ,ab209847, 1:1000 for WB);
Rabbit anti-H1N1 Influenza A virus Nucleocapsid protein, (Abcam,ab104870, 1:1000 for WB, 1:100 for flow cytometry);
Mouse anti-Influenza A Virus Hemagglutinin, (Abcam ,ab8262, 1:1000 for WB).

Validation

Vendor validation information in available online for the following antibodies:
Galanthus Nivalis Lectin (GNL), Fluorescein (Vector Labs, Cat# FL-1241-2)
Goat anti-rabbit IgG (H+L) antibody conjugated to fluorescein isothiocyanate (Sigma–Aldrich, Cat#AP307F);
Rabbit anti-β-actin (Invitrogen, Cat# PA5-78715);
Rabbit anti-Influenza A H1N1 NA (Invitrogen, Cat# PA5-23363);
Rabbit anti-DYKDDDDK Tag (Invitrogen, Cat# PA1-984B);
Mouse anti-Phosphomannose Isomerase (Invitrogen, Cat# MA5-25979);
Mouse anti-HIF1alpha (Invitrogen, Cat# MA1-516);
Mouse anti-GAPDH (Invitrogen, Cat# MA1-16757);
Goat anti-Rabbit IgG (H+L) (Invitrogen, Cat# 31460);
Goat anti-Rabbit IgG (H+L), Alexa Fluor®488 conjugate (Invitrogen, Cat# A11008);
Goat anti-Mouse IgG (H+L) (Invitrogen, Cat# 31430);
Goat anti-Mouse IgG (H+L), Alexa Fluor®594 conjugate (Invitrogen, Cat# A11005);
Rabbit anti-Hexokinase II (Abcam, Cat# ab209847);
Rabbit anti-H1N1 Influenza A virus Nucleocapsid protein, (Abcam Cat# ab104870);
Mouse anti-Influenza A Virus Hemagglutinin, (Abcam, Cat# ab8262).

Eukaryotic cell lines

Policy information about cell lines and Sex and Gender in Research

Cell line source(s)

A549(CCL-185),Calu3(HTB-55™), RD(CCL-136), MDCK(PTA-6503), VeroE6(CRL-1586™) were obtained from ATCC.
Huh7(0403™) were obtained from JCRB®. U251(09063001) were obtained from Sigma-Aldrich.
The Human Small Airway Epithelial Cells (SAEc) were obtained from the Lonza company (Catalog #: CC-2547).
The Human Nasal Epithelial Cells (hNEc) were obtained from the promocell company (Catalog #: C-12620).
The Bronchial/Tracheal Epithelial Cells (hBTEc) were obtained from the ATCC company (ATCC PCS-300-010).

Authentication

The commercially available cell lines have not been authenticated after receiving them.

Mycoplasma contamination

All cells were tested negative for mycoplasma contamination.

Commonly misidentified lines (See ICLAC register)	No commonly misidentified cell lines were used in this study.

Animals and other research organisms

Policy information about studies involving animals; ARRIVE guidelines recommended for reporting animal research, and Sex and Gender in Research

Laboratory animals	Mice aged 6-8 weeks were kept in biosafety level 2 or 3 housing and given access to standard pellet feed and water ad libitum, with individual ventilation with 65% humidity and ambient temperature ranging between 21-23 degree celsius with 12-hour-interval day/night cycle for housing and husbandry. In terms of gender, male K18-hACE2 transgenic mice, female BALB/c mice, and female IFNα/βR-/- (A129) mice were used.
Wild animals	The study did not involve wild animals.
Reporting on sex	For all in vivo experiments, same gender mice were randomized into different experimental groups.
Field-collected samples	The study did not involve samples collected from the field.
Ethics oversight	The use of animals has complied with all relevant ethical regulations and was approved by the Committee on the Use of live Animals in Teaching and Research of The University of Hong Kong

Note that full information on the approval of the study protocol must also be provided in the manuscript.

Plants

Seed stocks	Nil
Novel plant genotypes	Nil
Authentication	Nil

Flow Cytometry

Plots

Confirm that:

☒ The axis labels state the marker and fluorochrome used (e.g. CD4-FITC).

☒ The axis scales are clearly visible. Include numbers along axes only for bottom left plot of group (a 'group' is an analysis of identical markers).

☒ All plots are contour plots with outliers or pseudocolor plots.

☒ A numerical value for number of cells or percentage (with statistics) is provided.

Methodology

Sample preparation	Cells were detached from the culture plate using 1ml of enzyme-free dissociation buffer (Sigma) and fixed by adding 1ml of 4% formaldehvde for 24h at room temperature. Cells were washed once by with Perm/Wash buffer (BD) and stained for the relevant antibody and then secondary antibody. After 1h incubation at room temperature, cells were washed in phosphate buffered saline supplemented with 2mM EDTA once and resuspended in 200 microliter for analysis.
Instrument	The BD FACSCanto™ II Cell Analyzer
Software	FlowJo(v10.0.7)
Cell population abundance	Flow cytometry is used to quantify virus-infected cells but did not sort them. Therefore the question for post sort abundance is actually irrelevant.
Gating strategy	All cells were selected in a plot in which FSC-H was plotted versus SSC-H. Within this gate, positive cells were quantified.

☒ Tick this box to confirm that a figure exemplifying the gating strategy is provided in the Supplementary Information.
